# A Four-Port MIMO Cylindrical DRA with High Isolation in Ultra-Compact Size for WLAN Applications

**DOI:** 10.3390/mi14091671

**Published:** 2023-08-27

**Authors:** Xue-Ping Li, Jun-Fei Ji, Chang-Jiao Duan, Qian-Qian Sun, Wei Li, An-Xue Zhang

**Affiliations:** 1 College of Electronic and Electrical Engineering, Henan Normal University, Xinxiang 453600, China; jfji2021@126.com (J.-F.J.); cjduan2020@126.com (C.-J.D.); 18836225231@163.com (Q.-Q.S.); 2 Henan Key Laboratory of Optoelectronic Sensing Integrated Application, Henan Normal University, Xinxiang 453600, China; 3 Henan Engineering Laboratory of Additive Intelligent Manufacturing, Henan Normal University, Xinxiang 453600, China; 4 School of Information and Communications Engineering, Xi’an Jiaotong University, Xi’an 710049, China; anxuezhang@mail.xjtu.edu.cn

**Keywords:** cylindrical dielectric resonator antenna (DRA), decoupling, multiple-input-multiple-output (MIMO)

## Abstract

A novel ultra-compact four-port multiple-input-multiple-output (MIMO) cylindrical dielectric resonator antenna (DRA) with improved isolation is proposed for WLAN applications in this paper. The antenna is originally radiated with the assistance of two different excitation mechanisms to generate decoupled orthogonal modes. To further diminish the coupling field and improve the isolation, a suitable U-shaped slot is created on the common ground plane. Two additional rectangular slits are also etched to adjust the impedance matching of other ports. To better reveal the operating mechanism of the decoupling scheme, the common mode (CM) and differential mode (DM) impedance analysis methods between DRA ports are presented. The etched U-shaped slot can tune the impedance of CM and DM to be consistent to realize the decoupling. The antenna is simulated, fabricated, and tested to verify the decoupling mechanism. The results demonstrate that the isolation between ports 1 and 2 is enhanced from 5 dB to 23 dB, and other ports exhibit low coupling of better than 12 dB. Moreover, the antenna with the full size of 30 × 30 × 8.1 mm^3^ can be used either as a four-port DRA with a bandwidth of 300 MHz or as a two-port DRA with a bandwidth of 700 MHz, at a center frequency of 5.6 GHz.

## 1. Introduction

Nowadays, multiple-input-multiple-output (MIMO) technology, which makes use of multiple antennas on the transmitter and receiver sides, is capable of drastically improving channel capacity and reliability without requiring additional spectrum or transmission resources [1,2]. Considering these advantages, it has become one of the most critical technologies in current wireless local area networks (WLANs). However, some practical issues, such as the size and mutual coupling between antenna elements, are the primary challenges in realizing a high data rate and large capacity [1]. Thus, the design of low mutual coupling for multiple antennas with a compact size is suggested to allow the MIMO system to realize a large capacity.

Dielectric resonator antenna (DRA) has drawn great attention from antenna designers for wireless communication systems thanks to its notable characteristics, such as no conductor loss, small size, high radiation efficiency, and easy excitation [3]. Additionally, the excited multiple modes within the DRA could be utilized to produce multiple decoupled ports, which can be regarded as independent transmitting or receiving branches in a MIMO antenna system paradigm [4,5,6,7,8]. Therefore, the multifunctionality of DRA can reduce the demand for multiple antenna array elements and allow one element DRA to be used as a MIMO antenna [9,10,11,12,13]. However, as two or more antenna elements are arranged closely in a finite-size environment, the performance of the whole antenna system will be deteriorated undesirably because of its inferior isolation [14].

Up to now, plenty of decoupling methods have been introduced to alleviate the mutual coupling between antenna elements to achieve high isolation and acceptable DRA performance, such as parasitic elements [15,16], detected ground structures (DGSs) [17,18], metamaterial [19,20,21,22], and orthogonal modes induced by hybrid feeding mechanism [23,24]. An L-shaped dual-band MIMO DRA, which is excited by two microstrip line bending coupling slots and a cylindrical air gap to achieve good isolation of 17 dB, was introduced for LTE applications in [10]. Another dual-band MIMO design with the cylindrical DRA for the bands of DCS and WLAN is demonstrated in [9]. This antenna possesses good isolation thanks to the DRA-created orthogonal modes in the DRA. Triple-port MIMO cylindrical DRA-generated TM_01δ_ and HEM_12δ+1_ modes were designed in [11]. Das et al. presented a six-port and back-to-back four-port MIMO DRA-created gradient FSS as partially reflecting surfaces for the use of WLAN [21,22], where the isolation of the system can be realized over 20 dB and the size of the four-port antenna was 112 × 112 × 31.6 mm^3^. In [23], a spatially independent eight-port box-formed MIMO DRA was designed with the isolation of better than 20 dB and a size of 52.4 × 52.4 × 25 mm^3^ by adopting the exciting technology of orthogonal mode and spatial decoupled radiation mode. More recently, specific higher-order modes have been proposed to realize the decoupling between the two antennas, which can improve the isolation around 30 dB in [25]. Moreover, polarization quadrature [26] and orthogonal circularly polarized modes [27] have also been widely applied. As there is no need to introduce additional decoupling structures, it is beneficial for the realization of miniaturization design of the MIMO antenna [25,26,27]. Although the majority of the previous studies have presented acceptable decoupling results, these designs generally exhibit complicated configurations and manufacturing processes, large volume, high profile, and limited decoupling bandwidth.

In this work, we propose a simple and effective method to improve the isolation of the quadruple port in the compact MIMO DRA system. Firstly, four feed ports are integrated into one dielectric resonator in orthogonal form. Then, by introducing a suitable U-shaped slot, the original poor isolation of 5 dB between two ports can be improved to 33 dB across the whole band. Finally, two additional rectangular slits are etched for further tuning the impedance matching of other ports and the coupling. To better understand the inner mechanism of the presented decoupling scheme, the common mode (CM) and differential mode (DM) impedance analysis method is developed in MIMO DRA for the first time. One antenna prototype is then constructed and measured, and some analyses are applied to verify the presented decoupling scheme of this compact DRA.

The present article is arranged as follows. Section 2 presents the antenna engineering process and the physical principle of solving the coupling problem among MIMO DRA ports based upon the CM and DM. Section 3 illustrates the results of the simulation and measurement to further validate the proposed design. Moreover, the comparison with other existing works is listed to state the advantages. In Section 4, the paper is concluded.

## 2. Antenna Geometry and Design

### 2.1. Antenna Configuration

**Figure 1:**
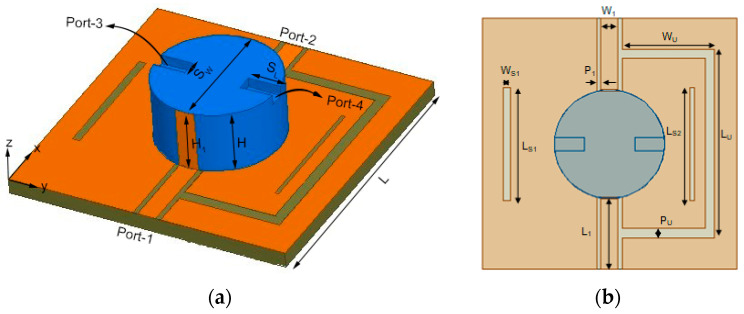
Antenna configuration of the proposed DRA. (**a**) 3D view. (**b**) Top view.

The configuration of the presented design is illustrated in Figure 1; two parts are included. One part is a cylindrical DR with relative dielectric constant of 9.8 fabricated on FR4 substrate. The other part is the feeding structure containing coplanar waveguides (CPWs) (ports 1 and 2) and probe feeds (ports 3 and 4). A U-shaped slot and two additional rectangular slits are created on the ground. The optimum dimensions are given in Table 1. The detailed engineered procedure of the MIMO DRA is exhibited as follows.

### 2.2. Decoupling Mechanism and Antenna Design

**Figure 2:**
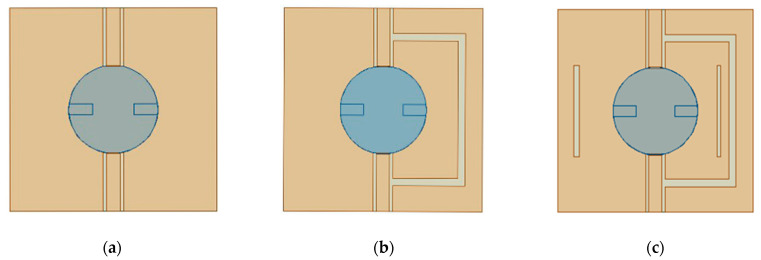
Design evolution of the proposed antenna. (**a**) Antenna 1. (**b**) Antenna 2. (**c**) Final antenna.

The design evolution of the proposed four-port MIMO DRA can be divided into three steps, as shown in Figure 2. In the beginning, the multifunctionality of the DRA could omit the need for multiple antenna array elements and enable the usage of one-element DRA as a MIMO antenna. Antenna 1 is proposed as the basic model with four ports fed in a dielectric resonator unit and two different feeding mechanisms. To realize decoupling between port 1 and port 2, Antenna 2 introduces a U-shaped slot between the two ports based on the effective impedance analysis method of CM and DM, so that the mutual coupling effect can be totally eliminated by making the CM and DM impedance the same. Considering the requirements of improving the impedance matching of port 3 and port 4, two rectangular slits perpendicular to the coaxial line are etched on the ground plane. Based on this process, the final structure can be obtained.

**Figure 3:**
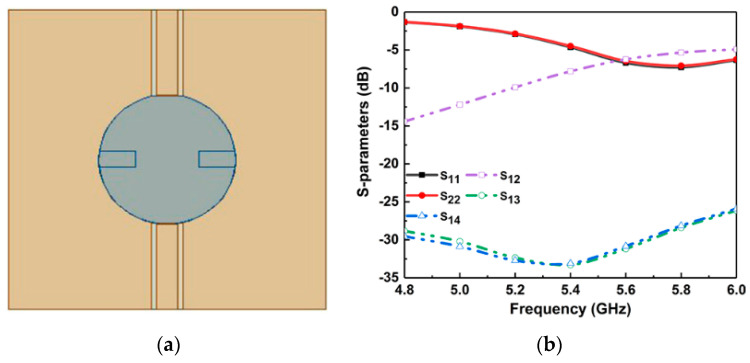
(**a**) Antenna 1. (**b**) Simulated S-parameters of Antenna 1.

As shown in Figure 3a, port 1 (port 3) and port 2 (port 4) are symmetric with each other; that is, S_11_ = S_22_, S_33_ = S_44_, S_13_ = S_24_, and S_23_ = S_14_. In the following, port 1 is used as an example to explain the decoupling between port 1 and other ports. We can clearly observe from Figure 3b that perfect isolation (S_13_ < −30 dB) is achieved between port 1 and port 3 without additional operations because orthogonal modes (${\mathrm{HE}}_{11\delta }^{y}$ and ${\mathrm{HE}}_{11\delta }^{x}$) can be produced at DR by orthogonal positions for the two ports. In addition, ports 1 and 2 are both excited by CPWs, while the other ports are excited through coaxial probe. Moreover, Figure 3b also exhibits that both port 1 and port 2 have poor return loss and reverse transmission. The two symmetrically placed ports with the same excitation pattern and close spacing cause strong coupling, causing the antenna element to not work independently. Therefore, the design focuses on the decoupling structure between the symmetrical ports and close ports, and the corresponding method is listed in detail in the following. 

As is known, the purpose of antenna decoupling is to make S_21_ = 0. Based on [28,29], we can know that, in a symmetrical and reciprocal dual-port antenna structure, the decoupling problem between two ports in theory can be equivalent to tuning the corresponding CM and DM impedances to be consistent.

(1) $$\left|S_{cc11}-S_{dd11}\right|=2\left|S_{21}\right|$$

where $S_{cc11}$ is the reflection coefficients of CM and $S_{dd11}$ represents that of DM.

**Figure 4:**
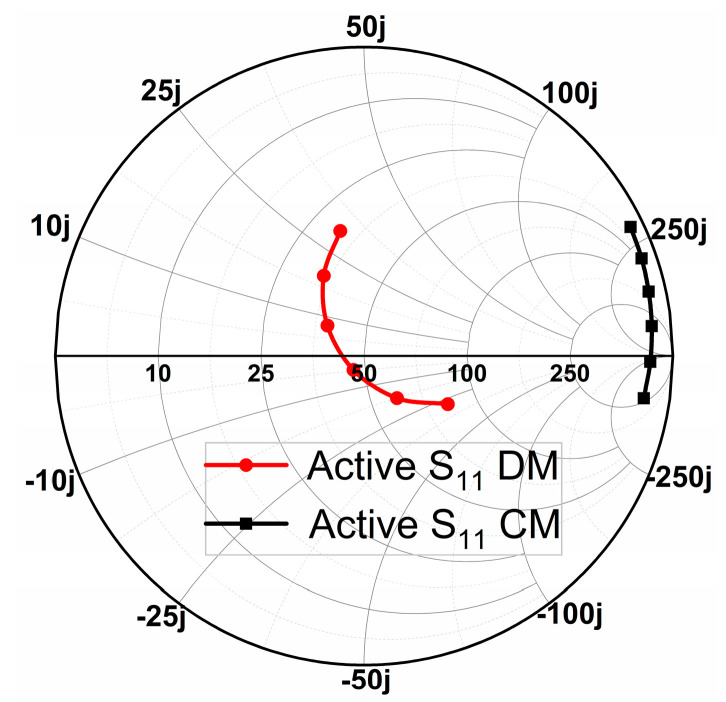
Simulated Smith chart of S_11_ for Antenna 1 with CM and DM (frequency ranges: 5.4–5.9 GHz).

Thus, we can exploit CM and DM impedance analysis to study and mitigate the mutual coupling between different ports of Antenna 1. As shown in Figure 4, the active impedance of the CM can be obtained by simultaneously exciting port 1 and port 2 with in-phase signal. On the contrary, if port 1 and port 2 are simultaneously excited with out-of-phase signal, the active impedance of the DM is realized. Furthermore, we can see that the value of CM impedance is greater than 50 Ω, while that of DM lies around 50 Ω. The discrepancies of impedance between CM and DM can cause strong mutual coupling for these ports, which implies poor isolation. Thus, it is crucial to tune the corresponding CM and DM impedances to be consistent.

**Figure 5:**
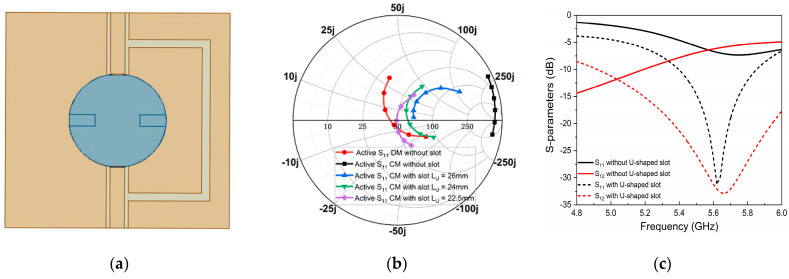
(**a**) Antenna 2. (**b**) Simulated Smith chart of S_11_ for Antenna 2 with CM and DM by change in U-shaped slot length (frequency ranges: 5.4–5.9 GHz). (**c**) Simulated S-parameters of Antenna 1 and 2.

To realize a similar impedance for CM and DM, one U-shaped slot was added at the port of Antenna 1 to serve as a parallel LC resonant circuit (see Figure 5a), and the corresponding antenna is called Antenna 2. Figure 5b demonstrates the active S_11_ for Antenna 2 with CM and DM by changing the slot length Lu. It is clearly seen that the diminution of slot length can induce the CM impedance gradually to match 50 Ω and the similarity can be found with an optimized length of 22.5 mm. Meanwhile, the DM impedance basically remains around 50 Ω with the change in slot length, and it is not discussed in this paper for brevity. S_11_ and S_12_ with and without a decoupling U-shaped slot are shown in Figure 5c. We can find that both the impedance matching and isolation are promoted under the use of a U-shaped slot. The isolation can be improved from 5 dB to 33 dB.

**Figure 6:**
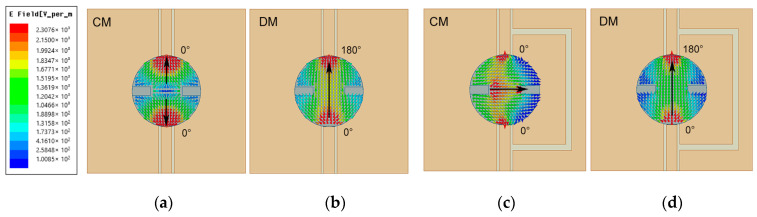
Vector electric field at 5.6 GHz with (**a**) CM and (**b**) DM excitation for Antenna 1 and (**c**) CM and (**d**) DM excitation for Antenna 2.

Figure 6 demonstrates the vector electric field distributions with and without inserting a U-shaped slot in the viewpoint of CM/DM for an intuitive comparison. Without the inserted decoupling slot, as depicted in Figure 6a,b, port 1 and port 2 are perfectly symmetrical, and the CM excitation can produce equal electric fields in magnitude and antiphase for two ports, causing the electric fields to cancel each other out. However, the strength of the electric field exhibits an increased trend for the DM excitation, which is induced by the identical electric field for the two ports. Thus, the energy in the DM is much higher than that in the CM, leading to the coupled field at the ports not completely offsetting. Figure 6c,d exhibits the electric field of Antenna 2 under the status of CM and DM. When DM is excited, the electric field distributions are similar to that of Antenna 1, as shown in Figure 6b,d. It is further verified that the introduction of the U-shaped slot has little effect on DM. However, as the CM is excited, after inserting the U-shaped slot, the electric field generated on the upper surface of the DR can change from reverse to in-phase, as shown in Figure 6a,c. Thus, the electric field strengths of CM and DM are changed to be the same as each other, which can effectively reduce the coupling between the two ports. In other words, the U-shaped slot creates a new coupling path that can directly cancel out the original coupling at the port.

**Figure 7:**
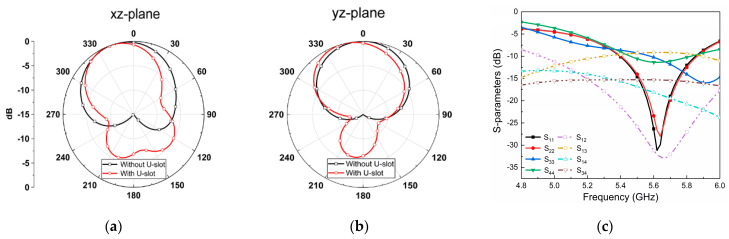
Simulated 2D radiation pattern with and without the U-shaped slot when port 1 is stimulated for the (**a**) xz-plane and (**b**) yz-plane. (**c**) Simulated S-parameter between the ports in Antenna 2.

To better demonstrate the improvement in isolation caused by the introduction of the U-shaped slot, Figure 7 exhibits the 2D radiation patterns of excitation port 1. We can find that the maximum radiation direction of Antenna 2 will be deflected in the opposite direction of the x-axis, which can reduce the overlap of space fields between the two ports and further improve the isolation. Although the back lobe is slightly larger by slotting on the ground plane, the improvement in isolation is considerable. As can also be seen from the S-parameters shown in Figure 7c, high isolation of 33 dB is achieved, but the inserted U-shaped slot breaks the orthogonal pattern between port 1 and port 3, deteriorating the performance of the two originally highly isolated ports, but within the usable range [30].

**Figure 8:**
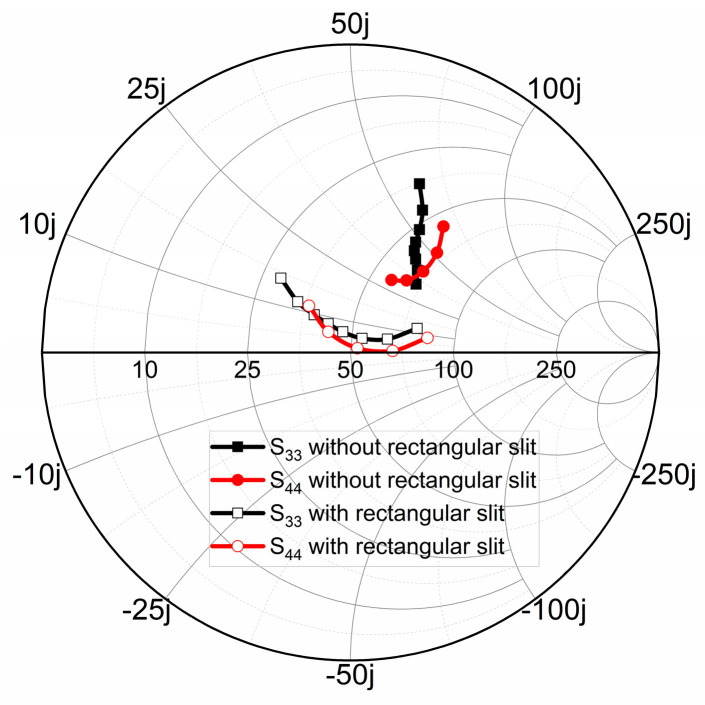
Simulated Smith chart of S_33_ and S_44_ for Antenna 2 and the proposed antenna (frequency ranges: 4.9–5.6 GHz).

**Figure 9:**
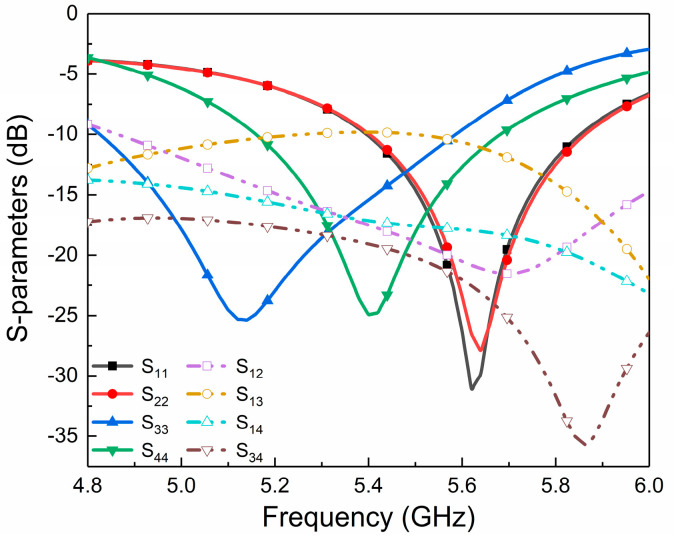
Simulated S-parameters for the proposed antenna.

For ports 3 and 4, two coaxial lines are symmetrically located on both sides of the pro-posed DRA and inserted laterally into the feed, which can induce good isolation between the two ports. To improve the performance of impedance matching for each port and enable different ports to cover different frequency bands, the quarterwave rectangular slit perpendicular to the coaxial line is also etched on the ground plane. This design provides two additional degrees of freedom to the matching network. Based on the common bandwidth of four ports and composite applications of 5G and WLAN, port 3 and port 4 can achieve strong resonance at low frequencies by adjusting the length and position of the rectangular slit. The Smith chart in Figure 8 shows S_33_ and S_44_ with and without the inserted rectangles slits. We can observe that both port 3 and port 4 of the proposed antenna can achieve the matching impedance of 50 Ω. As can also be seen from the simulated S-parameters exhibited in Figure 9, both the reflection coefficients and isolation for the two ports are improved. Although it has a slightly negative impact on the decoupling between port 1 and 2, the reflection coefficient and isolation of all ports are in a good state as a whole.

**Figure 10:**
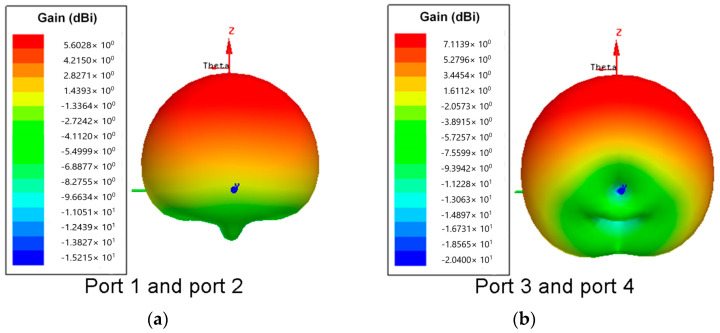
Simulated 3D radiation pattern fed by different ports for the MIMO DRA. (**a**) Port 1 and port 2 with a frequency of 5.6 GHz. (**b**) Port 3 and port 4 with a frequency of 5.4 GHz.

In addition, each port of the proposed antenna can be used separately or both together. In other words, when four ports are used separately, each port is individually connected to the feed port, and the other three ports are connected to the load to form a four-port MIMO antenna. When two ports are used together, two symmetrical ports (port 1 (port 3) and port 2 (port 4)) are fed differentially, and the other two ports are connected to the load. It is notable that the orthogonal modes in the DRA are excited by the two symmetric ports simultaneously, and their 3D radiation patterns are demonstrated in Figure 10. In such a case, the gain of the DRA can be up to 5.6 dBi and 7.1 dBi, respectively, which is higher than the gain of a single-port feed owing to the improved radiation pattern.

## 3. Results and Discussion

**Figure 11:**
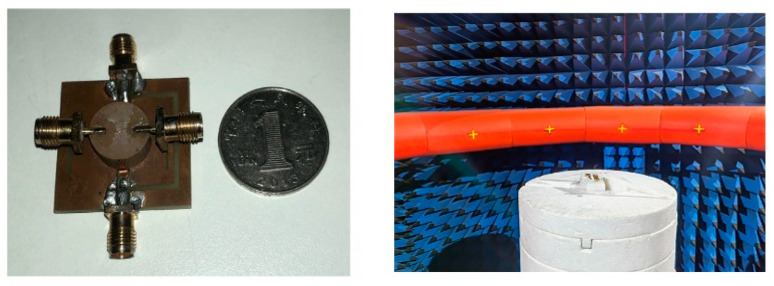
Photograph of the designed MIMO DRA and test setup in an anechoic chamber.

**Figure 12:**
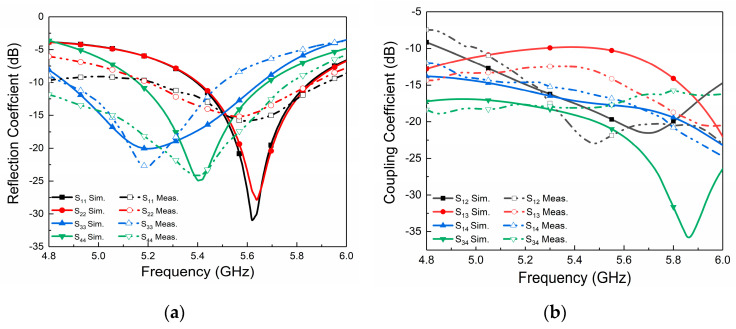
Simulated and measured (**a**) reflection coefficients and (**b**) isolation of the proposed DRA.

To demonstrate the effectiveness of the proposed four-port MIMO cylindrical DRA, an actual antenna prototype with a size of 30 × 30 × 8.1 mm^3^ is simulated, constructed, and measured (see Figure 11). Owing to the symmetry of their structures, we also take port 1 as the example to investigate the measured S-parameters. Figure 12 demonstrates the simulated and measured S-parameter results. The measured impedance bandwidths for antenna ports are 5.2–5.9 GHz (12.6%), 5.2–5.9 GHz (12.6%), 4.85–5.5 GHz (12.5%), and 4.8–5.75 GHz (18%), respectively. Figure 12b demonstrates that the isolation within the effective bandwidth is retained below 15 dB, but, owing to the compact size and multiple ports, the port of S_13_ exhibits a slight performance degradation, with the isolation being better than 12 dB. However, lower isolation has also been widely applied in 5G terminals [30,31,32].

**Figure 13:**
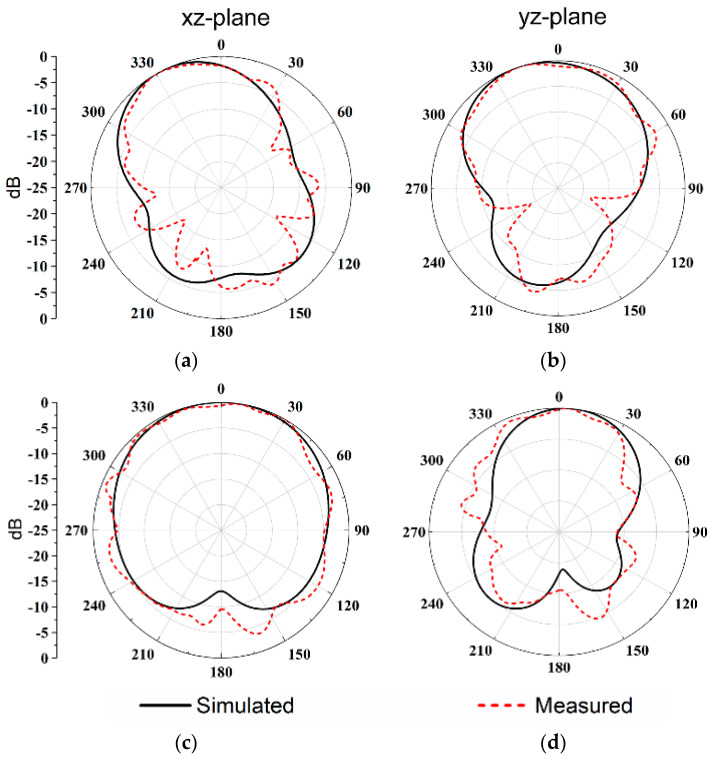
Simulated and measured 2D radiation pattern for the proposed DRA with a frequency of 5.6 GHz for (**a**) port 1 xz-plane and (**b**) yz-plane, frequency of 5.4 GHz for (**c**) port 4 xz-plane and (**d**) yz-plane.

Figure 13 demonstrates the simulated and measured normalized 2D radiation patterns for the presented antenna excited from port 1 and 4. We can see that the measurement and simulation are in accordance with each other. It is worthy to note that the proposed antenna system is symmetrical, which can make the radiation pattern exhibit symmetry in the xz-plane as well as in the yz-plane when fed by port 1 and 2. The gain of the four ports at the center frequency is 5.4 dBi, 5.4 dBi, 4.5 dBi, and 4.5 dBi, respectively. 

**Figure 14:**
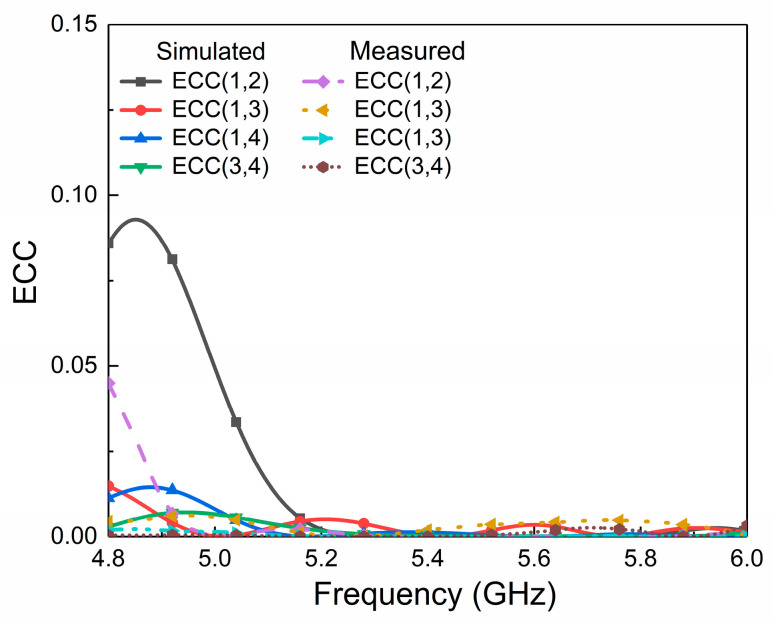
Simulated and measured ECCs of the proposed antenna.

To evaluate the diversity characteristics and MIMO properties of the designed antenna, Figure 14 shows the envelope correlation coefficient (ECC) between antenna ports. The results show that the ECC between all ports in the common bandwidth is less than 0.004, indicating that a low correlation between the ports and good diversity performance can be expected.

For a better comparison, the performances between the proposed MIMO cylindrical DRA and other existing studies are summarized in Table 2. As seen, the antennas in [10,13,15,25] have a lower number of ports and larger size than the antennas proposed in this paper. In addition, the decoupling method using metal strips in [15] can reduce mutual coupling between close DRA and achieve high isolation, but with only 4.8% bandwidth. The gain in [10,26] is lower. Meanwhile, the cylindrical air-gap introduced in [10] also possesses a complicated manufacturing process. In [22], the antenna is a back-to-back structure with decoupled scheme FSS, which can achieve higher isolation, but also has the disadvantage of a larger size and narrower bandwidth. In [13,25], multiple decoupling modes or higher-order modes are excited without additional decoupling structures, reducing the cost but inevitably increasing the size. To sum up, the antenna has excellent performance with an ultra-compact size, low profile, and high isolation, and our decoupling scheme also possesses the advantage of a simple structure with a tiny footprint, allowing its potential application in size-limited devices and terminals.

## 4. Conclusions

An ultra-compact cylindrical MIMO DRA with four ports on one dielectric resonator with improved isolation for the use of WLAN is presented. The isolation between two ports is raised by utilizing the excitation of orthogonal mode and suitable connecting etched slots. Then, a prototype antenna is made and tested; the detailed design guide is demonstrated to state the decoupling technology and verify the proposed method. The results indicate that the proposed decoupling scheme can improve the isolation between ports 1 and 2 from 5 dB to 23 dB across the whole band of 5.2–5.9 GHz, and other ports also exhibit low coupling of better than 12 dB. Moreover, the ECCs between the ports are also lower than 0.004 in the common bandwidth. The presented DRA antenna possesses a compact structure, high isolation, and good diversity performance, and it can be realized with the full dimensions of 30 × 30 × 8.1 mm^3^. The proposed antenna has potential applications as either a quadruple-port MIMO antenna or a dual-port antenna with operating bandwidths of 300 and 700 MHz, respectively.

## Figures and Tables

**Table 1:** Optimized numerical values of the designed antenna. Units: mm.

Variable	Value	Variable	Value	Variable	Value
L	30	W_1_	2	W_S1_	0.8
D	13	H_1_	6.5	L_S1_	13.5
H	6.5	L_1_	8.5	L_S2_	13.5
P_1_	0.5	P_U_	1.1	S_L_	3.5
L_U_	22.5	W_U_	11	S_W_	1.6

**Table 2:** Comparison with other existing works.

Ref.	No. of Ports	Decoupling Technique	Antenna Size	Operating Frequency Range (GHz)/Fractional Bandwidth	Max. Gain (dBi)	Isolation (dB)
[10]	2	Cylindrical air-gap	0.735λ_0_ × 0.735λ_0_ × 0.159λ_0_ 100 × 100 × 23.6 mm^3^	1.71–2.05, 2.5–2.7 (18%, 8%)	5.5	17
[13]	3	Polarization and pattern	1.79λ_0_ × 1.79λ_0_ × 0.445λ_0_ 56.6 × 56.6 × 14.09 mm^3^	9.12–9.84 (7.6%)	8.1	20
[15]	2	Metal strips	1.818λ_0_ × 1.818λ_0_ × 0.254λ_0_ 20 × 20 × 2.785 mm^3^	27.25–28.59 (4.8%)	9.9	24
[22]	4	FSS	1.965λ_0_ × 1.965λ_0_ × 0.316λ_0_ 112 × 112 × 16.6 mm^3^	5.15–5.35 (3.8%)	7.2	22
[25]	2	Higher-order modes	0.86λ_0_ × 0.52λ_0_ × 0.49λ_0_ 50 × 30 × 28.5 mm^3^	5.01–5.41 (7.7%)	7.8	20
[26]	4	Polarization and pattern	0.75λ_0_ × 0.68λ_0_ × 0.21λ_0_ 66 × 60 × 18.6 mm^3^	3.22–3.72 (14.4%)	4.2	15
This work	4 or 2	U-shaped slot	0.54λ_0_ × 0.54λ_0_ × 0.145λ_0_ 30 × 30 × 8.1 mm^3^	5.2–5.5, 5.2–5.9 (5.6%, 12.6%)	7.1	12–23

## Data Availability

Not applicable.
